# ﻿*Chimonobambusapleiacantha* (Poaceae, Bambusoideae), a new species from southeast Sichuan, China

**DOI:** 10.3897/phytokeys.241.120017

**Published:** 2024-05-02

**Authors:** Dan-Dan Zhao, Jin-Jun Yue

**Affiliations:** 1 Anji Bamboo Exposition Garden of Zhejiang Province, 313300, Anji, China Anji Bamboo Exposition Garden of Zhejiang Province Anji China; 2 Research Institute of Subtropical Forestry, Chinese Academy of Forestry, 311400, Hangzhou, China Research Institute of Subtropical Forestry, Chinese Academy of Forestry Hangzhou China

**Keywords:** Bambuseae, *Chimonobambusa*, new species, taxonomy

## Abstract

*Chimonobambusapleiacantha*, a newly-identified species of the genus *Chimonobambusa* Makino from southeast Sichuan, China, is meticulously described and illustrated herein. It is morphologically similar to *Ch.tuberculata*, but differs in having 4-angled internodes, thicker wall to 4.5–8.5 mm, more reclinate and elongated root thorns to 5–8 mm long, culm internodes with three grooves and two longitudinal ridges on the branch-bearing side, persistent culm leaves, densely brown-purple setose at the bottom of culm leaf sheaths together with sheath scar, developed foliage leaf fimbriae, 6–8 on each shoulder, ca. 3–8 mm long, abaxially white pubescent foliage leaf blades. Phenologically, new shoots usually appear in September to October. In the light of these key morphological and phenological characteristics, *Ch.pleiacantha* was identified as a new species of the genus *Ch.* Makino which is different from published species of this genus.

## ﻿Introduction

*Chimonobambusa* Makino, as a quite valuable genus in Bambusoideae, has garnered considerable attention. The yield of the plants is relatively high and the bamboo shoots have a high nutritional value. Usually, the bamboo shoots taste delicious and can be used for making a variety of cuisines. Furthermore, several species offer substantial economic benefits, notably due to the off-season development of new shoots in autumn, which presents a distinct seasonal advantage over other bamboos prevalent in the market. In recent years, in order to deeply implement the principles of the seed industry, enhance the collection, preservation, organisation and identification of bamboo germplasm resources and strengthen the foundation of breeding, many botanists have been continuously investigating the resources of the genus *Chimonobambusa*. In the process, some new taxa have been discovered. Yi et al. (2005) found a new species *Ch.pingshanensis* Yi et J. Y. in Pingshan, Sichuan; later, Cao et al. (2022) also published a new species *Ch.wumengensis* A. J. Cao in World Bamboo and Rattan Vol. 20. To date, over 30 species and subtaxa have been published in the genus (Shi et al. 2022).

*Chimonobambusa* Makino is characterised by rhizomes amphipodial, internodes terete or 4-angled, basal nodes often with a ring of sparse or dense root thorns, culm leaf auricles minute or absent, culm leaf blade reduced to 1 cm, narrow and inflorescence fully bracteate, 1–3 single pseudospikelet racemes loosely fasciculate, subtended by gradually enlarged bracts, spikelets sessile, rachilla disarticulating, glumes usually 1–3, frequently one subtending a bud, lodicules 3, membranous, stamens 3, filaments free, ovary ellipsoid, style 1, stigmas 2 or 3, plumose. It is mainly distributed in the south-western mountainous regions of China with its core distribution spanning Yunnan, Guizhou and Sichuan Provinces and a few species of the genus extend to the southern slopes of the Qinling Mountains or along the south-eastern coast. The optimal elevation range for their growth is from 500 to 2600 m (Xue and Yi 1982). Situated in the heart of southwest China, Sichuan Province boasts a warm climate, conducive to the proliferation of diverse bamboo resources. Recent studies and comprehensive surveys indicate that the bamboo resources in Sichuan Province involve about 14 species and one variety of *Chimonobambusa* Makino bamboos. The plants of the genus often form extensive pure forests (Yi et al. 2008).

Xingwen County is located in the south-eastern zone of Yibin City, Sichuan Province and is characterised by its distinctive climate, topography, hydrology and soil conditions, which have fostered the development of numerous high-quality bamboo species of the genus (Sun 2023). In mid-October 2023, in order to systematically explore the germplasm resources of *Chimonobambusa* Makino in Xingwen County, we made several expeditions of the local bamboo base of the genus. During our return from Jiusi Town to Xingwen County, we encountered an unidentified bamboo species. At that time, it was in the intermediate stage of shooting, displaying its complete morphological characteristics, which indicated this species belongs to *Chimonobambusa* Makino. The culm leaf sheaths of this unknown bamboo initially are yellowish-white, tinged with purplish-red towards the apex and the abaxial side with yellowish tuberculate setose, which is similar to *Ch.tuberculata*, but can be easily distinguished by its 4-angled internodes, thicker wall to 4.5–8.5 mm, slanted downward and curved at the apex root thorns to 5–8 mm long, culm internodes with three grooves and two longitudinal ridges, persistent culm leaves, densely brown-purple setose at the base of culm leaf sheaths together with sheath scar, developed foliage leaf fimbriae to 3–8 mm long, foliage leaf blades with abaxially white pubescent. In the light of investigations and morphological comparisons, this bamboo was identified as a unique and previously undescribed species of *Chimonobambusa*, which is meticulously described and illustrated in this paper.

## ﻿Materials and methods

The plant material of the new species was collected from Jiusi Township, Xingwen County, Sichuan Province, on 14 October 2023. Morphological features were observed and documented during the field survey. The height of the plant was measured using a hand-held laser rangefinder, while other significant quantitative traits for taxonomic classification, such as bamboo diameter, internode length, leaf length and leaf width, were measured using a tape measure and Vernier caliper. The specimens were stored in the Herbarium of Anji Bamboo Exposition Garden, Zhejiang, China and Research Institute of Subtropical Forestry, Chinese Academy of Forestry. Terminologies in this paper referring to the relevant descriptions follow Wen (1994), Xue and Zhang (1996) and Clark and Cortés (2004).

## ﻿Taxonomy

### ﻿ Chimonobambusa pleiacantha

Taxon classificationPlantaePoalesPoaceae

D. D. Zhao & J. J. Yue sp. nov.

EB338BAE-086B-59EC-A062-28982666B366

urn:lsid:ipni.org:names:77335475-1

Figs 1 , 2 , 3 , 4

#### Diagnosis.

*Chimonobambusapleiacantha* resembles *Ch.tuberculata*, but can be effortlessly distinguished by having 4-angled internodes (vs. terete), thicker wall to 4.5–8.5 mm (vs. 2–3 mm), more reclinate and elongated root thorns to 5–8 mm (vs. 2–4 mm), culm internodes with 3 grooves and 2 longitudinal ridges (vs. grooves obscure), persistent culm leaves (vs. deciduous), densely brown-purple setose at the base of culm leaf sheaths together with sheath scar (vs. without dense setae), conspicuously developed and persistent foliage leaf fimbriae to ca. 3–8 mm long (vs. spare and caducous) and foliage leaf blades with white pubescence on the abaxial side (vs. abaxially glabrous). (Table 1, Fig. 5).

**Table 1.** Differences in morphology between *Ch.pleiacantha* and *Ch.tuberculata*.

Characters	*Ch.pleiacantha*	*Ch.tuberculata*
Culm height	2–7 m	3–4 m
Culm diameter	0.5–4 cm	1.2 cm
Culm internode	internodes 4-angled, 10–20 cm long, flattened on branch-bearing side, with 3 grooves and 2 longitudinal ridges; wall 4.5–8.5 mm	internodes terete, 14–18 cm long, grooves indistinct on branch-bearing side; wall 2–3 mm
Culm intranode	nodes below mid-culm each with a ring of 4–10 root thorns, root thorns usually slanted downwards and curved at the apex, 5–8 mm long	nodes below mid-culm each with a ring of 4–12 straight root thorns, 2–4 mm long
Culm leaf	persistent	deciduous
Culm leaf sheath	initially yellowish tuberculate setose and densely brown-purple setose at the base together with sheath scar	initially yellowish-brown adnate setose
Foliage leaf fimbriae	6–8 on each shoulder, ca. 3–8 mm long, straight	sparse and caducous
Foliage leaf auricle	present or absent	absent
Foliage leaf blade	oblong lanceolate, 10–30 cm × 1.5–3 cm, abaxially white pubescent, secondary veins 5–7-paired, transverse veins indistinct	lanceolate, 19–25 cm × 2–3 cm, abaxially glabrous, secondary veins 6–9-paired, transverse veins distinct
New shoot	September to October	August to September
Habitat and distribution	on the hillside or under the forest at altitudes of 1000–1500 m, Xingwen County, southeast Sichuan	on the hillside at the elevation of 1300–1450 m, Yongshan County, Yunnan

#### Type.

China. Sichuan: Xingwen County, Jiusi Township, Fangbei Village. 28°6′19″N, 105°2′25″E, 1344 m alt., 16 October 2023, D. D. Zhao & J. J. Yue 23101 (holotype: Herbarium of Anji Bamboo Exposition Garden!; isotypes: Herbarium of Research Institute of Subtropical Forestry, Chinese Academy of Forestry!).

#### Description.

Shrubby bamboo. Rhizomes amphipodial. Culms 2–7 m tall, 0.5–4 cm in diam., erect; internodes 4-angled, 10–20 cm long, flattened on branch-bearing side, with 3 grooves and 2 longitudinal ridges; wall 4.5–8.5 mm. Intranodes 1–2 mm long, nodes below mid-culm each usually with a ring of 4–10 root thorns, root thorns usually slanted downwards and curved at the apex, rarely horizontally arranged, 5–8 mm long. Branches usually 3 per node, sometimes 4–5 on upper nodes. Young culms green, initially white pubescent and tuberculate hispid, later verrucose, rough; old culms dark green. Sheath scar initially with a densely upward brown-purple setose ring. Culm leaves shorter or longer than internodes, persistent; sheaths thinly papery or papery, yellowish-white when fresh and tinged with purplish-red towards apex, abaxially initially yellowish tuberculate setose and densely brown-purple setose at the base together with sheath scar, margins faint yellow ciliate; transverse veins indistinct; auricles and ligule both absent; fimbriae absent; blade erect, 0.5–1mm long. Foliage leaves 3–5 per ultimate branch; sheaths abaxially glabrous, upper margins ciliate; fimbriae developed, neatly arranged, 6–8 on each shoulder, straight, ca. 3–8 mm long; auricles present or absent; ligule ca. 0.5 mm; petiole 2–3 mm long; blades oblong lanceolate, 10–30 cm × 1.5–3 cm, abaxially greyish-green and white pubescent, secondary veins 5–7-paired, transverse veins indistinct, margins serrulate. New shoots from September to October are prone to browning after harvesting. Inflorescence unknown.

#### Vernacular names.

毛刺竹(Chinese name), máo cì zhú (Chinese Pinyin); 刺竹(Local common name), cì zhú (Chinese Pinyin).

#### Distribution and habitat.

**Figure 1.**
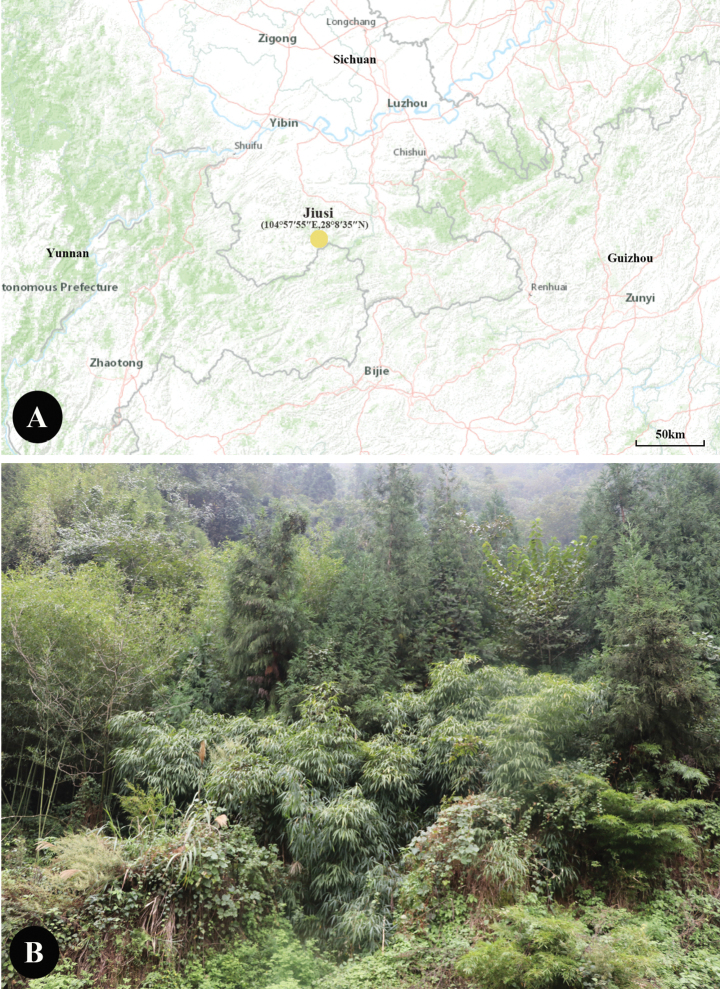
Distribution (A, the yellow circle showing its distribution area) and habitat (B) of *Ch.Pleiacantha*.

*Chimonobambusapleiacantha* was found in Xingwen County, southeast Sichuan Province, China, growing on the hillside or under the forest at altitudes of 1000–1500 m. (Fig. 1)

#### Conservation status.

*Chimonobambusapleiacantha* grows under mixed forests in the south-western mountainous area of Xingwen County with quite a number of populations involving large areas of both wild and cultivated groups. Additionally, the local government attaches great importance to the scientific development of bamboo resources, so it can be effectively protected. Thus, we tentatively assessed it as “Least Concern” (IUCN 2022).

**Figure 2.**
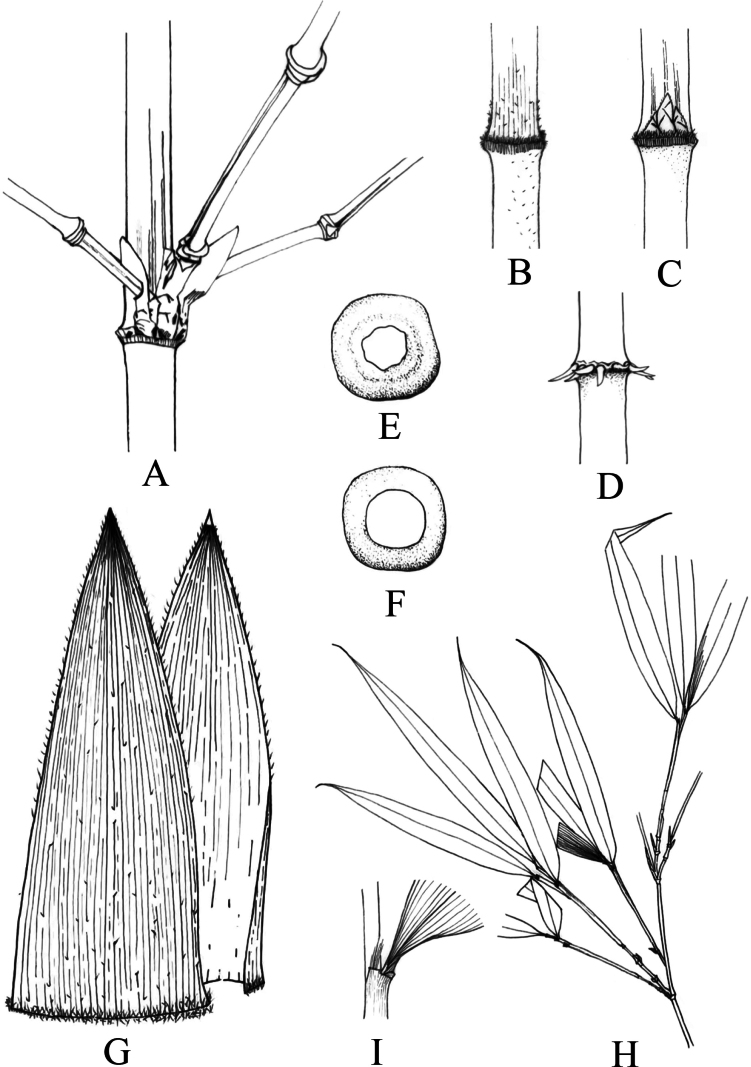
*Ch.pleiacantha***A** the branch of middle culm **B** the base of culm leaf sheath and the internode of young culm **C** the long oval buds on node **D** the decurved root thorns **E, F** cross section of old culm **G** culm leaf **H** ultimate branch and foliage leaves **I** details of foliage leaf sheath.

**Figure 3.**
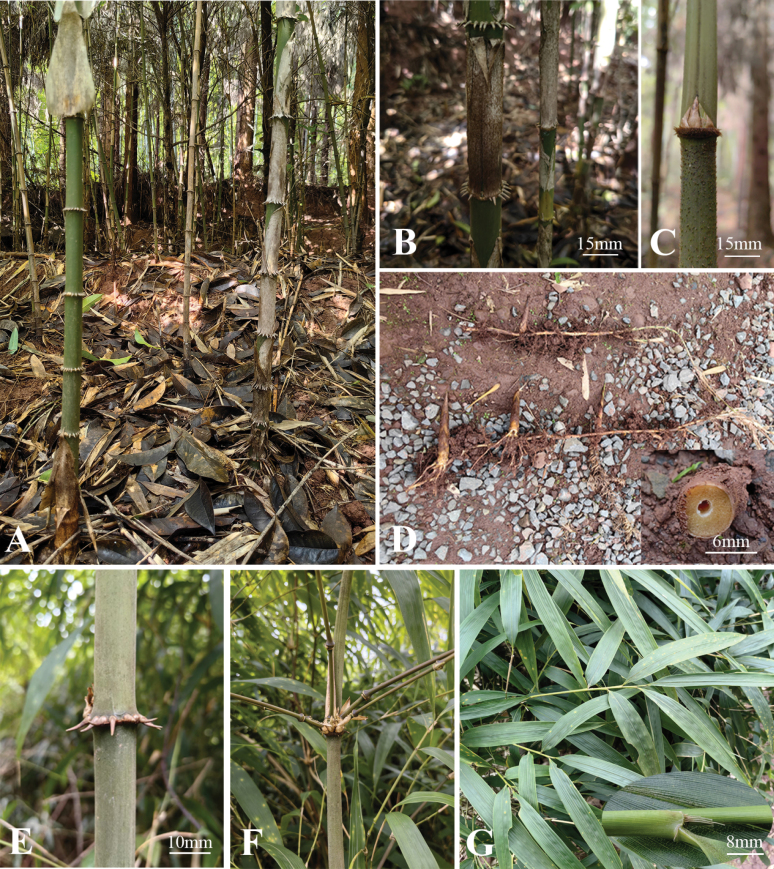
*Ch.pleiacantha***A** clump **B** internodes of lower culm, showing persistent culm leaves **C** a node, showing bud complements and setaceous sheath scar **D** rhizomes **E** decurved and elongated root thorns **F** branches of the mid-culm **G** foliage leaves in adaxial view, showing blades, sheaths and fimbriae.

**Figure 4.**
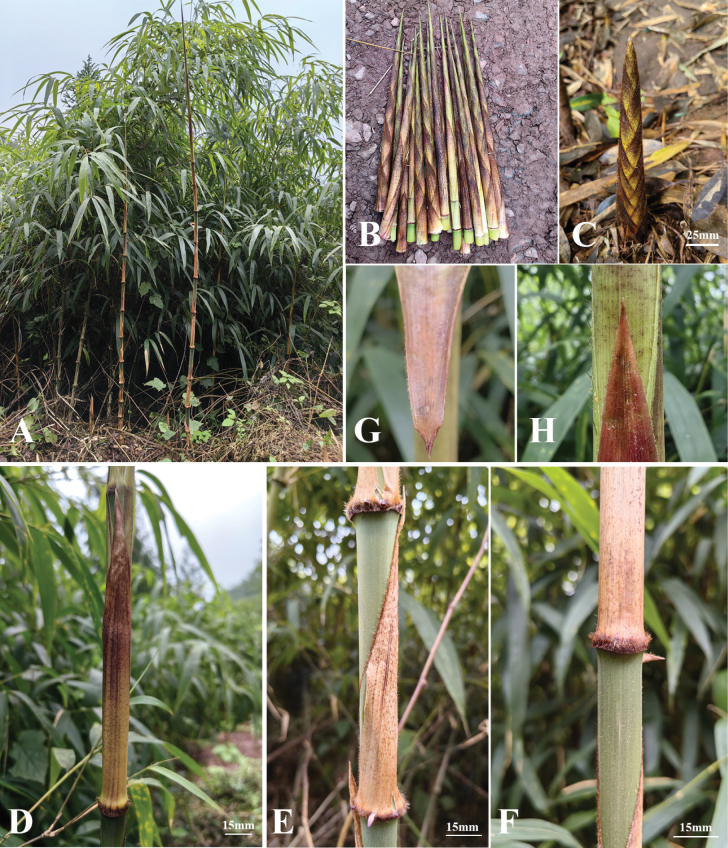
*Ch.pleiacantha***A–C** shoots, showing fresh culm leaf sheaths yellowish-white, apically purplish-red **D** the abaxial side of culm leaf sheath, with yellowish tuberculate setae **E** the lateral side of culm leaf sheath, showing margins with faint yellow cilia **F** the base of culm leaf sheath, with densely brown-purple setae **G** upper part of culm leaf in adaxial view, showing absent ligule and auricles **H** upper part of culm leaf in abaxial view, showing shorter blade.

#### Phenology.

Bamboo shoots developed in September and October.

#### Etymology.

The specific epithet “pleiacantha” refers to the back of the bamboo sheaths with many spines.

**Figure 5.**
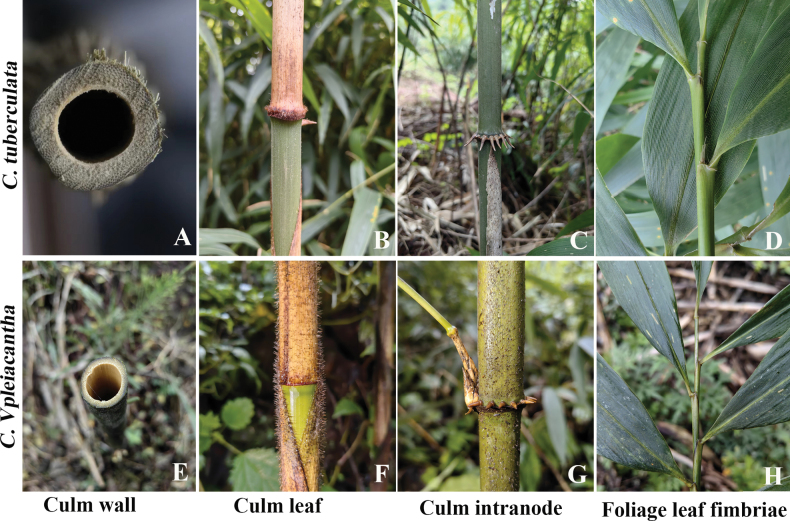
Comparisons of partial key morphological characters between *Ch.pleiacantha* and *Ch.tuberculata*.

## ﻿Discussion

*Chimonobambusa* Makino was first published as a new genus by Makino from Japan in 1914, which involved two species, *Ch.quadrangularis* (Fenzi) Makino and *Ch.marmorea* (Mitford) Makino; meanwhile, the flowers of *Ch.marmorea* (Mitford) Makino were described and selected as the type species of *Chimonobambusa* (Wen 1994). Since then, the taxonomic work of this genus has been continuing. In the 1940s, Y. Z. Keng published a new genus *Oreocalamus* Keng and designated *O.szechuanensis* as the type species of *Oreocalamus*, which was collected in China (Keng 1940). However, because its morphological characteristics and ecological habits are compatible with those of the genus *Chimonobambusa*, Xue and Yi (1982) believed that it is inapposite to recognise *Oreocalamus* Keng as a separate genus. In the 1980s, Hsueh and Yi (1980) published another new genus, *Qiongzhuea* Hsueh et Yi and designated *O.szechuanensis* as its type species, Nonetheless, this genus is similar to *Chimonobambusa* in terms of inflorescence structure, so whether this genus is valid has been questioned (Chen and Lu 1994). Based on these, Wen (1994) divided *Chimonobambusa* Makino into three sections: Ch.sect.Chimonobambusa, Ch.sect.Oreocalamus (Keng) Wen & Ohrnberger ex Ohrnberger and Ch.sect.Qiongzhuea (Hsueh & Yi) Wen & Ohrnberger ex Ohrnberger. Subsequently, Ch.sect.Qiongzhuea was separated and restored to a separate genus by Xue and Zhang (1996); Yi et al. (2008) and Shi et al. (2021) also accepted the taxonomic opinion of them, but did not approve the division of sections. Although the inflorescence and fruit of *Ch.pleiacantha* are unknown, rhizomes amphipodial, internodes 4-angled, young culms green, initially white pubescent and tuberculate hispid, later rough after verrucae falling, nodes below mid-culm with a ring of root thorns, branch usually three per node, sometimes 4–5 on upper nodes, culm leaf sheaths persistent, thinly papery or papery, shorter or longer than internodes, blade erect, 0.5–1mm long, with typical taxonomic characteristics of the genus *Chimonobambusa*. Through the collection of originally published literature and investigation into the origins of the genus *Chimonobambusa*, we ultimately identified *Ch.pleiacantha* as a new species, which is differ from all other known species of the genus, based on the morphological comparisons, but did not make the division for the time being.

By reviewing the original published literature and investigating the origins of the genus *Chimonobambusa*, we discovered that *Ch.pleiacantha* is similar to *Ch.tuberculata*, but exhibits distinct differences in several aspects. During the investigation, we found that the information of the collection site of the type specimen of *Ch.tuberculata* was incorrectly documented, the correct location is “Shuidong Zi(水洞子), Xisha Township, Yongshan County, Yunnan Province” not “Xiaodong Zi(小洞子), Xisha Township, Yongshan County, Yunnan Province”. Furthermore, the morphological characteristics of *Ch.tuberculata* are described as “internodes terete, grooves indistinct on branch-bearing side; young culms initially with brown dense tuberculate hispid, later rough after verrucae falling; nodes below mid-culm each with a ring of 4–12 root thorns; culm leaves deciduous, papery or thickly papery, abaxially initially with yellowish setose, becoming black verrucose; 3 or 4 leaves per ultimate branch; blade lanceolate, 19–25 × 2.2–3 cm, secondary veins 6–9-paired, transverse veins distinct. Inflorescence unknown. New shoots Aug-Sep (Xue and Li 1987)”. During the investigation, our findings also revealed that the sheath scar of *Ch.tuberculata* lacks a densely brown-purple setose ring and foliage leaf fimbriae are sparse and deciduous. These characteristics which we found are aligned closely with the descriptions in Flora of China, Vol. 9(1) by the Committee on Flora of China, Chinese Academy of Sciences (1996).

## Supplementary Material

XML Treatment for Chimonobambusa pleiacantha

## ﻿Additional information

### Author ORCIDs

Dan-Dan Zhao https://orcid.org/0009-0005-5155-7006

Jin-Jun Yue https://orcid.org/0000-0001-9606-6394

### Data availability

All of the data that support the findings of this study are available in the main text.
